# A Study on Digit Sucking Among Children Presented to a Tertiary Care Paediatric Clinic in Sri Lanka

**DOI:** 10.7759/cureus.13306

**Published:** 2021-02-12

**Authors:** Vijayakumary Thadchanamoorthy, Kavinda Dayasiri

**Affiliations:** 1 Clinical Sciences, Faculty of Health Care Sciences, Eastern University, Batticaloa, LKA; 2 Paediatrics, Base Hospital, Mahaoya, Mahaoya, LKA

**Keywords:** digit sucking, thumb, malocclusion of teeth, dental surgeon

## Abstract

Background

Sucking habits are common among children, especially in early childhood. There is, however, controversy about its prevalence probably due to under-reporting. The objective of this study is to analyze the demographic factors, reasons for clinic attendance, and treatment options chosen by parents and health care providers to control digit sucking.

Material and methods

A retrospective cross-sectional study was conducted including 82 children presented to the paediatric clinic, Teaching Hospital, Batticaloa, with digit sucking over a period of three years from November 2017 to October 2020. The data was analyzed using SPSS version 19.0 (IBM Corp., Armonk, NY, USA).

Results

Eighty-two children were enrolled in this study. The majority (n=29, 35.4%) of them presented between seven months to two years, and very few (n=3, 3.6%) presented after five years. Female children outnumbered males (50: 32). Only 24 (29.3%) mothers had been working and away from home during the daytime whilst the rest were housewives and were available to their children most of the time of the day. About 70 (85.4%) children had been given exclusive breastfeeding while the rest had either formula alone or formula with breastfeeding during the first six months of their age. Around 62 (75.6%) children had used their right hand for sucking. Sixty-six children (80.5%) had been using the thumb. Most children (n=56, 68.3%) were sucking since birth. The most common reason (n=68, 82.9%) for clinic attendance was that parents were scared about the future occurrence of mal-alignment or malocclusion of teeth. Thirty-one parents (37.8%) did nothing to stop digit sucking while 25 (30.5%) applied bitter oil and the majority tried pacifier without success. All children were referred to the dental surgeon for further management of digit sucking and amongst them, 78 children (95.1%) attended the appointment. All 78 children had been reassured that the habit eventually disappears before permanent teeth will appear and explained that digit fixing therapy would be offered for persisting habitual digit sucking. Four children did not visit the dental department.

Conclusions

The majority of children presented to the clinic were below three years and females outnumbered males in all age groups. A greater number of children had been using their right hand and thumb. Most of them had the habit of sucking since birth and most of the parents anticipated mal-alignment of teeth due to consequences of sucking. None of them succeeded in stopping the habit of digital sucking with various remedies performed at home by their parents.

## Introduction

Digit sucking is the most common oral fixation among children [1]. Digit sucking is a habit performed by frequent repetitive action due to either conscious or unconscious motor activities [2-4]. With each repetition, initially, it becomes less conscious, and eventually, it becomes either subconscious or unconscious motor activity [3]. The prevalence varies according to their demographic, racial, and socioeconomic status [5]. Either thumb or any other finger is used for sucking to various extents (6). This action is thought to satisfy both nutritive and non-nutritive needs of children according to their situation [6, 7]. Non-nutritive sucking habits (NNSH) are the more common form that persists up to 36 months of life [8, 9]. Nevertheless, it might prolong and can cause occlusive, aesthetic, and psychological changes [10]. Children who are breastfed for at least nine months have a low incidence of non-nutritive sucking habits, although it may vary in different areas of the world at different periods [11, 12]. Further, the incidence of digit sucking has been more among infants up to one year of age and the incidence decreases with age and spontaneous termination is often noticed by the age of four to five years before permanent teeth appear [13]. The authors performed a retrospective cross-sectional study of 82 children who presented to the paediatric clinic with the complaint of digit sucking over a period of three years at Teaching Hospital Batticaloa.

## Materials and methods

A retrospective cross-sectional study was conducted among 82 children who attended paediatric clinic in the professional unit of Teaching Hospital Batticaloa over a period of three years from November 2017 to October 2020. All children who attended the paediatric clinic during this three years period were included in this study. Relevant information was retrieved from their clinic records and the questionnaires were completed by investigators. The only limited information that was documented consistently in clinic records were included in the study. The records which had insufficient data were omitted in the study. Information retrieved included age, sex, mother’s occupation, the reason for clinic attendance, duration of sucking, sidedness of hands used for sucking, digits used, types of feeding up to first six months, remedies opted by parents, previous medical treatment, and response to medical treatment. The data was analyzed for descriptive statistics using SPSS version 19 (IBM Corp., Armonk, NY, USA). Permission from the Director, Teaching Hospital Batticaloa, was obtained for the study, but it did not warrant ethical clearance as it was a record-related study.

## Results

The majority of children (81.8%) had been below three years with the predominant proportion being between seven months to two years. Generally, female children (n=50, 61%) outnumbered male (n=32, 39%) in all age groups except in the group of children aged between 3-5 years. Only 24 (29.3%) mothers had been working whilst the rest (n=58, 70.1%) were housewives. Seventy children (85.4%) had been given breastfeeding while the rest had either formula alone or formula with breastfeeding up to the first six months of life. Table 1 shows the distribution of age and sex in the study population with digit sucking.

**Table 1:** Distribution of age and sex

Age	Female	Male	Total	
			Number (N)	Percentage (%)
Birth to 6 month	12	6	18	22
7 months to 2 years	18	11	29	35.4
2+ to 3years	14	6	20	24.4
3+ to 5 years	4	8	12	14.6
5+ to 8 years	2	1	3	3.6
Total	50	32	82	100

Majority (n=62, 75.6%) of them used right hand while rest (n=20, 24.4%) were using left hand. About 80.5% (n=66) of children had been using the thumb and 12.2% (n=10) had been using both index and middle finger together. Only 7.3% (n=6) were using the middle three fingers. Table 2 demonstrates the pattern of hand and finger preferences of the study population with digit sucking. 

**Table 2:** Pattern of hand and finger preferences for digit sucking

Types	Right side	Left side	Total	
			Number (N)	Percentage (%)
Thumb	52	14	66	80.5
Index and middle finger	6	4	10	12.2
Middle three finger	4	2	6	7.3
Total	62	20	82	100

Most children had been sucking since birth (n=56, 68.3%) whilst 29.3% were digit sucking since five to seven months of life. Only 2.4% (n=2) of children started to suck after seven months.

The reason for clinic attendance was analyzed as illustrated in Table 3. The majority (n=68, 82.9%) of parents anticipated the occurrence of mal-alignment of teeth with the growth of their children. 26.8% (n=22) of parents thought the poor feeding was due to the digit sucking.

**Table 3:** Reason for clinic attendance

Reasons	Number	Percentage (%)
Poor feeding	22	26.8
Vomiting of foods	11	13.4
Snoring	05	6.1
Bad smell from mouth	06	7.3
Worry about mal-occlusion of teeth	68	82.9
Recurrent paronychia	08	9.8
Recurrent diarrhea	06	7.3
Difficulty in writing	05	6.1

As demonstrated in Table 4, 37.8% (n=31) of parents did nothing to stop the habit of digit sucking in their children. 30.5% (n=25) applied bitter oil to stop it. Almost 51.2% (n=42) tried pacifiers to stop the habit with no success. All were referred to the dental surgeon for further management for digit sucking by either the Out Patient Department (OPD) or General Practitioner (GP). Among them, 78 (95.1%) children visited and had been reassured as the habit eventually will disappear before permanent teeth appeared.

**Table 4:** Measures offered by parents

Measures	Total	
	Number (N)	Percentage (%)
None	31	37.8
Apply bitter oil	25	30.5
Tie the fingers	06	7.3
Hurt the finger	08	9.8
Pull the finger	05	6.1
Apply socks	07	8.5
Pacifier	42	51.2

## Discussion

Oral habits such as prolonged, intense, and continuous sucking of digits can interfere with normal growth and development of the jaws leading to malocclusion of teeth and also adversely affect the development of normal swallowing speech patterns [14]. The current study was done to evaluate all children presented to the paediatric clinic with the complaint of digit sucking. The study highlighted the demographic and clinical information of children with digit sucking. There were not any previously published Sri Lankan studies on children with digit sucking and this study might bring awareness about digit sucking and motivate them to do further studies related to this fact. Our study showed that the majority of children with digit sucking were below three years (n=67, 81.8%) and female (n=50, 61%). A different study was done in South Asia also had similar findings [13]. However, several other studies revealed it was more common in boys [15, 16] while other studies revealed no sex differences [17]. Therefore, the sex of the child may not be a strong factor to determine the habit of digit sucking.

Digit sucking after 36 months has been considered to be prolonged non-nutritive digits sucking and it was 20% according to a study by American Academy of Paediatric dentistry [6]. Similar findings were revealed in the current study (18.2%). Although digit sucking had been more common during early childhood, it regressed with age [13]. Our study revealed similar findings (3.6%).

A study among Brazilian children [14] revealed that children of working parents had a high incidence and breastfed babies had a lower incidence of thumb sucking. This could be due to early cessation of breastfeeding causing greater frustration to the child than the experience of no breastfeeding at all [18], although breastfeeding fosters close bonding between mother and child as well as emotional satisfaction between them [19]. Though it was very true, this finding contradicted our study in which most mothers were housewives (n=58, 70.1%) and most children were breastfed (n=70, 85.4%). Sri Lanka is a country that has high breastfeeding rates. Another reason may be that it is a different study setting with regard to demographic, racial, and socioeconomic conditions [5]. Most of the above-mentioned studies reported that the reduction of digit sucking was significantly related to increasing breastfeeding.

Majority of children used right hand (n=62, 75.6%) and thumb (n=66, 80.5%). This finding indicated that the dominant hand was used to suck by the majority. There were no studies that compared sidedness of hands and types of fingers used for sucking although most studies indicated thumb sucking as the most common finding in children with digit sucking [13-17]. Many children in our study had been sucking since birth (n=56, 68.3%), which might support the observations of the study that assessed the relationship between postnatal digit sucking and intrauterine sucking [20].

The reason for clinic attendance was also analyzed and the majority (n=68, 82.9%) of parents anticipated the occurrence of mal-alignment of teeth with the growth of their children. Twenty-two parents (26.8%) thought that poor feeding of both breastfeeding and solid food intake was due to the digit sucking behaviour, as revealed in our study. Three studies demonstrated occlusion of teeth due to digit sucking [12, 21, 22], while one other Indian study agreed with 43% of the children with malocclusion and 17% of them showing a reduction of breastfeeding [13]. In addition, our study revealed various other parental concerns such as frequent vomiting, bad smell from the mouth, snoring, recurrent paronychia, and recurrent diarrhoea, and felt that their child might find it difficult to write and end up with poor school performance. A study from India revealed mouth breathing in 63% of children with digit sucking [13].

Our study showed that approximately 37.8% of parents did nothing to control the habit of digit sucking whilst others had done various measures to control digit sucking although with no improvement, including applying bitter oil, tying of involved fingers by threads, hurting the finger while sucking, pulling off the finger during sucking and application of socks to prevent sucking. More than 50% of parents tried with pacifiers as a choice despite no improvement. A study done in India showed that the majority of their mothers were not aggressive in stopping the habits while others tried various measures such as applying gloves, nail polish, digit cap, etc. with no success [13]. Another study demonstrated that mothers did not want to stop that behaviour as their children were still young [23].

All children were referred to a dental surgeon for further management for digit sucking and of those, 78 (95.1%) children attended the dental clinic. Parents had been reassured that there was a little chance to have malocclusion if their child will not abandon the behaviour of digit sucking before four years or at least before the eruption of permanent teeth. However, it was difficult to convince many parents of digital fixing procedures to prevent digit sucking. A study in India revealed that only 38% visited the dentist and many parents were not initially convinced regarding the procedure of eliminating the digit sucking although eventually the parents could be reassured [13].

## Conclusions

Sucking behaviours were widespread in early childhood, and the majority of children with digit sucking were female children. The study highlighted that thumb and involvement of the right hand were predominant. Most children sucked from birth and this observation may show that digit sucking can have inherent properties from intrauterine sucking movements. It was challenging to convince the parents to accept the digital fixing procedures to prevent digit sucking in this culture. Although most parents were worried about the mal-occlusion of teeth the parents, however, they could be reassured that there was a little chance of having malocclusion if children can stop this habit before the eruption of permanent teeth.
